# K-D:rib dampens Hs 578T cancer cell chemoinvasion and proliferation

**DOI:** 10.1186/s12935-014-0077-9

**Published:** 2014-08-12

**Authors:** Luca Bruni, Adesola A Babarinde, Ida Ortalli, Simonetta Croci

**Affiliations:** 1 Neuroscience Department, Biophysics and Medical Physic Unit, University of Parma, Parma, Italy; 2 Valsè Pantellini Foundation, Calle Cervantes, 16/4 izda, Oviedo, Asturia, Spain; 3 INBB National Institute of Biosystems and Biostructures, Rome, Italy; 4 Department of Chemical Sciences, Olabisi Onabanjo University, Ago-Iwoye, Ogun State, Nigeria

**Keywords:** Hs 578T (HTB-126) human breast cancer cell line, Hs 578Bst (HTB-125) breast epithelial cell line, Chemotactic invasion assay, Potassium bicarbonate, D-ribose, Doubling Population time

## Abstract

**Background:**

Cancer cells, even in the presence of available oxygen, have a glycolysis enhancement. The “aerobic glycolysis” is known as the Warburg effect and it is considered one of the fundamental hallmarks of metabolic alteration during malignant transformation. A feature of many tumors is also a change into ions equilibrium, with particular reference to K^+^ intracellular concentration. Another hallmark in cancer is the reprogrammed chemotaxis pathways in favour of tumor cell dissemination.

**Results:**

The doubling population time of 5 mM K:D-rib treated Hs 578T (HTB-126 ® ATCC) cell line is reduce by 30% respect to the control. During the chemotactic invasion assay, the relative number of motile and invasive cells, counted inside the FBS-AGAR spot, shows a decrease with the maintenance of the treatment reaching the 25% after nine days. Hs 578Bst (HTB-125 ® ATCC) non-tumor cell line treated for nineteen days with 5 mM K:D-rib was split twice as well as the control. No morphological change was visible in the treated respect to untreated cells.

**Conclusions:**

We demonstrate that the synergic action of potassium bicarbonate and D-ribose has effect on Hs 578T cancer cell line proliferation reducing the cell cycle time. At 5 mM concentration, K:D-rib is able to modify the tumorigenic potential of human breast cancer cell line Hs 578T, interfering *in vitro* with the capability of Hs 578 T cell line to migrate under chemotactic stimuli. Despite this, K:D-rib solution, does not exhibit any appreciable toxicity as confirmed by the proliferation assay accomplished on Hs 578Bst cell line.

## Introduction

In this paper we investigate the effects of potassium hydrogen carbonate and D-ribose in water solution (K:D-rib) on the human breast cancer cell line - Hs 578T. We also present results on human Hs 578Bst cell line derived from normal breast tissue peripheral to an infiltrating ductal carcinoma, which was the source for Hs 578T. Our previous study [1] focused on the strong antioxidant effect of potassium ascorbate on red blood cell oxidation [2],[3] highlighted the ascorbate involvement as carrier of K^+^ inside the cells. The role of D-ribose in the energetic metabolism [4],[5] and its involvement into glycogen synthesis, as well as the importance of K^+^ into the cell physiology, are well known [6],[7]. It has been found that K^+^ is essential to fold and to stabilize G-quadruplex [8] with a strong relevance for telomeric structures of telomeres [9]. Agents that stabilize G-quadruplexes can act like anti-tumor agents [10]; so physiological K^+^ concentration is demanded for not cancer cell behaviour.

Our most recent work demonstrates that the 5 mM K:D-rib solution slows the replication of the Hs 578T cell line and has a cytostatic effect on canine carcinoma cell line (A72) [11]. The effects of potassium ascorbate together with D-ribose have been investigated on Beckwith - Wiedemann syndrome and in a case of Prader Willi syndrome and Costello syndrome, severe syndromes characterized both by a high oncological risk and by high ROS concentration [12]–[14].

It is well known that cancer cells show an increase in the consumption of sugars, in particular glucose, named “Warburg Effect” [15],[16]. The increase of glycolysis for energy production is a cancer hallmark that enables the tumor to growth rapidly, and it suppresses apoptosis up to anticancer therapy resistance [17]–[19].

Because of the hydrophilic nature of this sugar, cells use a number of glucose transport proteins divided in two families: the GLUT (facilitated glucose transporters) and SGLT (sodium/glucose). The GLUT transporters use the existing concentration gradients between the exterior and the interior of the plasma membrane to facilitate the sugar translocation. On the contrary, the SGLT proteins move the sugars into the cells against a concentration gradient, resulting in energy cost. The SGLT1 and SGLT2 proteins are able to exchange the sugars with Na^+^ through the plasma membrane to carry out this transport [20]. Recently has been also tested [21], with magnetic resonance imaging (MRI), the hypothesis that the physiological and biochemical changes associated with the proliferation of malignant tumors can cause an increase in the total concentration of Na^+^ in the tissues of patients with both benign and malignant cancer. All these evidences demonstrate that intracellular homeostasis for Na^+^ and K^+^ is no longer tightly regulated in cancer cells and also an alteration of Na^+^/K^+^ ATPase expression and activity has been shown in patients with cancer [22].

It can be stated [23] that the potassium channels or rather their over expression is involved both in the increased cell proliferation and in the apoptosis: a paradox to which the answer is difficult. In this context, the manipulation of K^+^ channel function and/or expression could be a difficult option for cancer treatment.

In the case of the cell line Hs 578T there is an overexpression of SGLT1, these data available on Pharma- cogenetics.UCSF.edu website and collected by *Novartis* are referring to the work of Huang Y. and colleagues [24]. This network database collects data of a project where investigators from diverse disciplines are conducting a series of integrated studies to elucidate the pharmacogenetics of membrane transport proteins.

Another hallmark in cancer is the reprogrammed chemotaxis pathways in favor of tumor cell dissemination [25]. Chemotaxis is the phenomenon by which the movement of cells is directed in response to an extracellular chemical gradient and it is the result of three separate steps: chemosensing, polarization and locomotion. Depending on the cell type and the microenvironment, migration can involve single unattached cells or multicellular groups. Tumor cells often express deregulated profiles of chemokine receptors; therefore chemokines and their receptors critically regulate cancer cell migration or contribute to cancer cell proliferation and survival. For example the CXCR4 chemokine receptor is highly expressed in human breast cancer cells. *In vitro* chemotaxis assays measuring the aptitude for the directed migration, are useful in the preclinical progression of potential therapeutics [26]. Several assay systems currently exist for the analysis of chemotaxis. However, most are costly, difficult to perform, susceptible to modified conditions during analysis, or difficult to multiplex.

The use of agarose, as medium through which chemoattractants can diffuse, has provided a simple and straightforward system to study cell motility [27].

In this work we calculate the Doubling Population (DP) time of Hs 578T cell line treated with K:D-rib respect to control. Then we test the effect of K:D-rib solution on cancer chemoinvasion of Hs 578T cell line.

The study will be completed estimating a grow rate of non-tumor breast epithelium cell line Hs 578Bst treated with K:D-rib compared with the untreated one.

## Materials and methods

### Cells and culture conditions

Hs 578T adherent human breast cancer cell line was obtained from American Type Culture Collection (Manassas, VA, USA). Hs 578T cells were maintained in low glucose Dulbecco’s Modified Eagle’s Medium (Sigma Aldrich) supplemented by: 10% Fetal Bovine Serum (Sigma Aldrich), 1% L-glutamine (Sigma Aldrich) and 1% penicillin-streptomycin (Sigma Aldrich). Hs 578T cell line was incubated at 37°C in humidified atmosphere with 5% CO_2_.

Hs 578Bst human mammary epithelial non-tumor cell line was purchased from American Type Culture Collection (Manassas, VA, USA). Hs 578Bst cell line was maintained in Hybri-Care Medium (ATCC) supplemented by 1.5 g/L sodium bicarbonate (Sigma). To make the complete growth medium, the following components were added to the base medium: 30 ng/ml mouse EGF (Gibco), 10% of Fetal Bovine Serum (Lonza), 1% penicillin-streptomycin (Sigma Aldrich). Hs 578Bst cell line was incubated at 37°C in humidified atmosphere with 5% CO_2_.

### Drugs and chemicals

Giemsa stain modified solution was bought from Fluka, paraformaldehyde and D-ribose were bought from Sigma Aldrich. KHCO_3_ was bought from BDH. 1 M K:D-rib solution was obtained by mixing 0.15 g of D-ribose and 0.3 g KHCO_3_ in distilled water, while CO_2_ blown away. The work solution was obtained by diluting the stock solution with distilled water.

12 mm diameter coverslips, coated with poli-L-lisyne, are bought from BD Italy.

### Cell proliferation assay

Cell proliferation is measured with two methods.

*Method 1*: Hs 578T cell line was seeded at the concentration of 20000 cells/ml in four-well plates; the volume of plated cell suspension was 500 μl. The cells have been treated with K:D-rib at the concentration of 5 mM for 96 hours. Every 24 hours the cells were fixed by PFA at 4%, stained by Giemsa stain solution. The entire well was pictured and all cells counted with the help of Image J program (Plugin – Cell Counter).

*Method 2*: Hs 578T cell line was seeded at 4000 cells/ml into seven 35 mm Petri dishes and treated for 14 days, at the concentration of K:D-rib of 5 mM. For the entire treatment duration, every 48 h one control dish and a treated one were fixed with PFA at 4% and stained with Giemsa stain solution. The cell counting was based on pictures acquired field by field, along two perpendicular axes crossing the Petri center. The counting was performed with the help of Image J program (Plugin – Cell Counter).

### Splitting number

Hs 578Bst cell line was seeded at the concentration of 4000 cell/ml and the cells treated with K:D-rib solution at the concentration of 5 mM. The duration of the experiment was 19 days and the cell medium was changed three times a week, according to our cell culture protocol. When the cells were approximately 90 - 100% confluent they were split 1:2. The splitting number is reported both for control cells and for 5 mM K:D-rib treated cells.

### Chemotactic invasion assay

This assay was performed following mainly the Wiggins’s protocol [28]. 0.05 g of low melting point agarose (Sigma Aldrich) was diluted with 10 ml PBS to obtain a solution of 0.5% agarose. It was heated up till boiling point and shaken to reach the complete dissolution, then removed from heat as the agar solution was completely melted. 90 μl of melted agarose were dropped into 1.5 ml tube, supplied with 10 μl of FBS (AGAR + FBS), as chemoactrant enhancer. In another tube 90 μl of agar alone (AGAR) were dropped. 10 μl of each agarose solution were pipetted on two 12 mm diameter coverslips coated with poly-L-lysine and placed in a 35 mm Petri dish. After that, the dishes were left 30 minutes waiting for AGAR cooling and for the right spot texture. Before the chemotactic invasion assay the cells were pre-treated with 5 mM K:D-rib solution for different time: 3, 5 and 7 days. At the end of each pre-treated time, 20000 cells/ml were plated into a dish containing the two agar spots and 5 mM K:D-rib solution. Another sample has be done using 5 day pre-treated cells, plated into a dish containing the two agar spots. In this case the treatment was suspended during the chemotactic assay. The duration assay was 48 h and at the end, each AGAR spot was photographed. For each spot the number of inner cells was counted and divided by the perimeter of each spot. The perimeter was calculated using Image J program. Cells not fully inside the spot were not counted. The same was done in case of control (cells without treatment) and the number of treated cells were normalized to the control.

### Statistical analysis

Data of cell proliferation assay are analysed with t-test to compare *method 1* and *method 2*. In details, the slope of linear regression of control data, obtained with *method 1*, is compared with the slope of linear regression of control data, obtained with *method 2*, using t-test for coupled data. Then the slope of linear regression of control data is compared with the slope of linear regression of treated data (both obtained with *method 2*) using, as well, t-test for coupled data.

## Results

### Doubling Population (DP) time on Hs 578 T

Two different counting methods on control Hs 578T cells (without treatment), have been used to establish a consistent way to calculate the DP time. Because it is well known that the cancer cells grow exponentially [24]*in vitro*, the number of cells was fitted with the following equation,

(1) $${\mathrm{In}}_{2}\;\left(N\right)=\frac{t}{d}+{\mathrm{In}}_{2}\;\left(N_{0}\right)$$

where N is the number of cells at t time, N_0_ (*method* 1) is the seeded cell number and d is the DP time.

In case *method 2*, 4000 cells/ml were seeded in a 35 mm Petri dishes. This time only the cells lying on two perpendicular axes crossing the Petri centre were counted. The experiment was extended to 14 days. In this case using equation 1 the obtained N_0_ value is proportional but not equal to the number of seeded cells. The d value stills the DP time.

**Figure 1:**
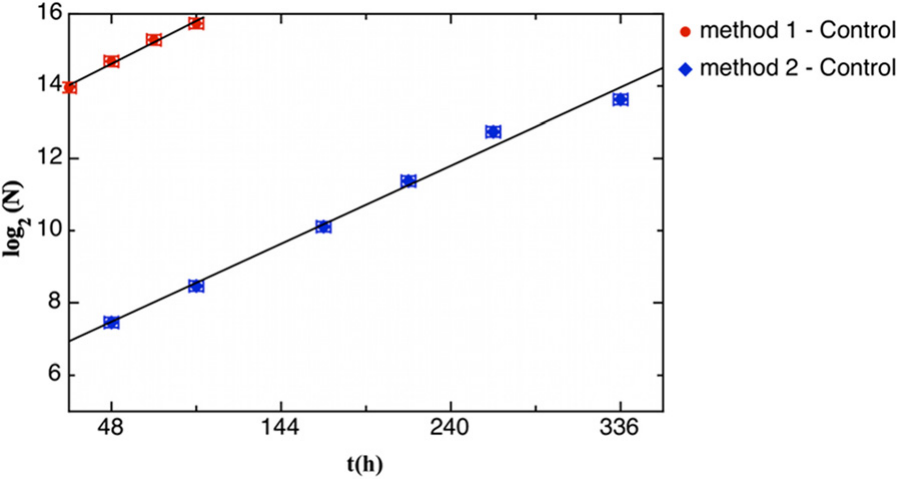
**Best fits with*method 1*and*method 2*.** Number of cells (N) is plotted against incubation time with *method 1* and *method 2*. The linear regressions have not significantly different slopes.

In Figure 1 are reported the best fits of data obtained using the two reported methods. In particular in case of *method 1* the best-fit procedure gives N_0_ = 11036 ± 153 cells and d = 40 ± 5 h. With this method we obtained a value of N_0_ consistent with the number of seeded cells (~10000).

With *method 2* we calculate the DP time that results d = 44 ± 1 h. We perform a t-test to compare the slopes of linear regression of the data obtained with *method 1* and *method 2*. The t-test gives a P = 0.63, therefore since the slopes are not significantly different, it is also possible to calculate a slope for all data. The pooled slope equals 0.0223 and consequently the control DP time is d_C_ = 44 ± 5 h.

**Figure 2:**
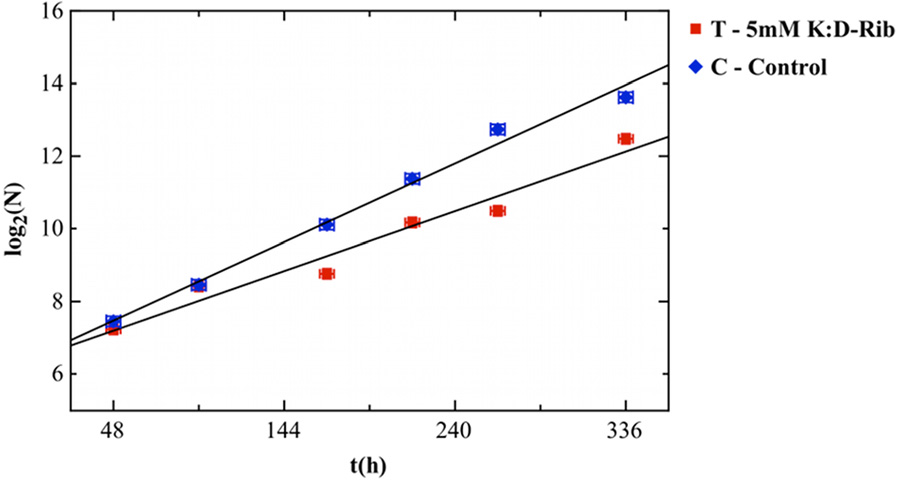
**Best fits of control and treated cells.** Number cells (N) as function of incubation time in case of control (blue) and 5 mM K-D:rib treated cells (red). The linear regressions have significant different slopes.

Since the two methods are not significantly different to calculate the DP time, we decided to use the *method 2* to calculate the DP time of the cells treated (T) with 5 mM K:D-rib. As reported in Figure 2 the linear regression of T data has a slope that is different to that one of the control (C) data: the DP time results to be d_T_ = 59 ± 2 h. To compare the slopes, we perform a t-test of the two data sets, resulting a value t = 3.114 (P = 0,0256) > t_c_ = 2,571 (P = 0.05). We can conclude that the difference between the slopes, and therefore the difference between the DP times of control and treated cells, is significant.

### Chemotactic invasion assay

**Figure 3:**
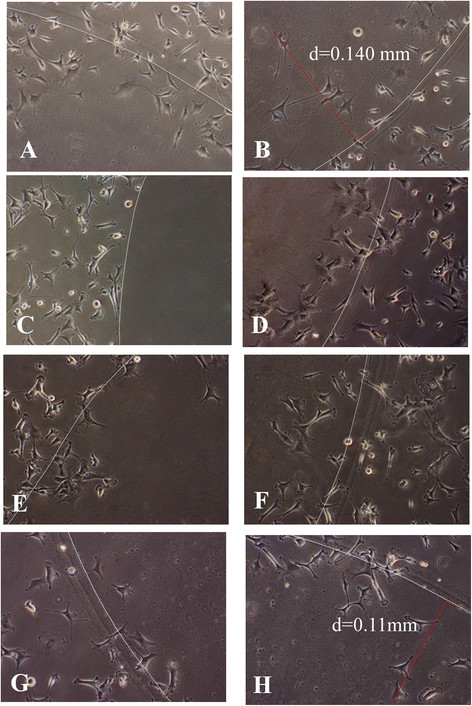
**Chemotactic invasion assay with AGAR - FBS spot.** On the left **(A)**, Hs 578T control cells into agar spot supplied with Fetal Bovine Serum (FBS). On the right **(B)**, is reported the maximum distance from the border (d = 0.140 mm) reached by control cells, in the spot radius direction. On the left **(C)** Hs 578T control cells on the border of AGAR spot without FBS. On the right **(D)** are reported Hs 578T cells relative to five days of 5 mM K:D-rib treatment: three pre-treatment days and two treatment days during the chemotactic assay. On the left **(E)** are reported Hs 578T cells relative to seven days of 5 mM K:D-rib treatment: five pre-treatment days and two treatment days during the chemotactic assay. On the right **(F)** are reported Hs 578T cells relative to the seven day K:D-rib treatment: seven pre-treatment days and two days of chemotactic assay without treatment. On the left **(G)** are reported Hs 578T cells relative to nine days of 5 mM K:D-rib treatment: seven pre-treatment days and two treatment days during the chemotactic assay. On the right **(H)**, is reported the maximum distance from the border (d = 0.11 mm) reached by cells, in the spot radius direction. The white line delimits the edge of the agar - FBS spot.

In Figure 3(A) is depicted the AGAR - FBS spot invaded by cancer cells, because of chemoactrant properties of Fetal Bovine Serum. Herein the high cell tumorigenic potential is shown. Hs 578T cell line comes into the FBS - AGAR spot from every point of spot edge. The white line draws the inner border of the spot.

In Figure 3(C) is reported the negative control of chemoinvasion assay: cells do not enter into the AGAR spot FBS free. AGAR alone has not the chemoactrant properties showed by AGAR - FBS. The cells reach the spot edge, they touch it but they are not able to get into the spot.

The treatment reported in Figure 3(D), relative to five days with 5 mM K:D-rib, consists on three pre-treatment days and two treatment days during the chemotactic assay. The cells come into the AGAR - FBS spot from any sides of the spot edge, as shown in the control test. The morphology has not been modified respect to the control.

Figure 3(E) illustrates migration and invasion after seven days of the treatment (five days of pre-treatment plus two treatment days during the chemotactic assay). The cell morphology looks like the control one. The Hs 578T cell line treated invades the AGAR - FBS spot from whole spot perimeter as described in the control. Figure 3(F) shows how the cells react without 5 mM K:D-rib during assay. The morphology is like to the control. This result is compared to that one get from 7 days of the K:D-rib 5 mM treatment (5 treatment days plus two assayed days with treatment).

Figure 3(G) highlights the effect of the longest treatment with 5 mM K:D-rib. In case of control, Figure 3(A), the maximum distance from the border reached by the cells along radius is 0,14 mm while in case of 9-day treatment; all the cells stay nearby the border, Figure 3(G). Only in the case reported in the Figure 3(H), the cells reach the distance of 0,11 mm. Although the treatment of the Hs 578 T cell line affects the chemoinvasion potential, the cell morphology looks like the control.

**Figure 4:**
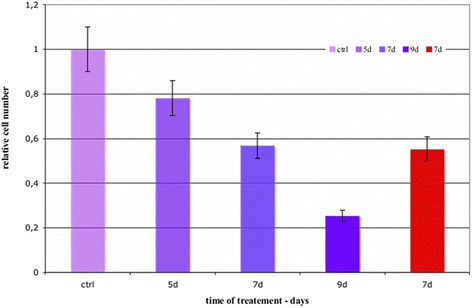
**Relative Hs 578T cell number.** Cells without treatment (ctrl) and cells after 5, 7, 9 days of treatment with 5 mM K:D-rib. 5, 7, 9 days of treatment include 48 hours of chemotactic invasion assay with treatment. The last column represents the relative cell number after 7 days of treatment but without treatment during 48 h of chemotactic invasion assay.

In the Figure 4 is plotted the relative number of motile and invasive cells counted inside the FBS-AGAR spots normalized to the control cell number and divided by the perimeter of the spot to avoid errors due to the different spot dimension.

The five day experiment (three treatment days plus 48 hours of treatment assay) and the seven day experiment (five treatment days plus 48 hours of treatment assay) show a relative cell number decrease with the maintenance of the treatment, compared with the control. The relative cell number of the nine day experiment (seven treatment days plus 48 hours of assay treatment) decreased to 25%.

When the Hs 578T cell line is treated for seven days and the chemotactic invasion assay is perform without treatment (red column), the relative cell number is greater than the nine day treatment (seven treatment days plus 48 hours of treatment assay). The relative cell number is the same (~55%) reached after the five days of treatment plus two treatment days during the chemotactic assay.

### K:D-rib affect Hs 578Bst proliferation

**Figure 5:**
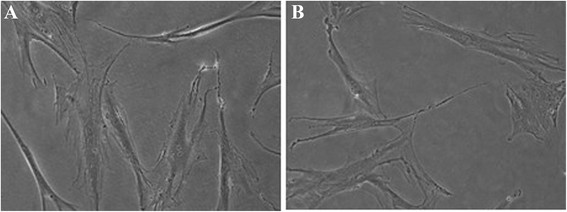
**Hs 578Bst cells.** On the left **(A)**, Hs 578Bst control cells at the day 12th; on the right **(B)** Hs 578Bst cells at day 12th of treatment with 5 mM K:D-rib.

Hs 578Bst cell line (human epithelial breast cancer) has been treated for nineteen days with K:D-rib water solution at the concentration of 5 mM. Both control and treated cells were split twice during the experiment. Figure 5(A) is relative to the control and in Figure 5(B) to the K:D-rib treated cells at twelfth day of the experiment. Analysing the morphology of the treated and untreated cells, it is clear that K:D-rib treatment do not change the morphology of Hs 578Bst cell line. Then, another cell proliferation experiment is performed to assay the effects of each K:D-rib component. Hs 578Bst cell line are treated with 5 mM of D-ribose, 5 mM of KHCO_3_ and 5 mM K:D-rib solution. The experiment duration was nineteen days, to compare it to previous Hs 578Bst cell line experiment. Not treated Hs 578Bst cell line has been split twice in each proliferation experiment as well as the treated. The treated cell with each separated components of K:D-rib solution do not display any morphological change.

## Discussion and conclusions

### Doubling Population (DP) time on Hs 578T

The assumption that the tumor cells grow exponentially is widely accepted [28] for early steady and for *in vitro* system. Because this hypothesis we calculate the duplication time assuming that this is equal to the cell cycle time. It is also well known that when these conditions are not fully satisfied, for example *in vivo* or in cell colony growing *in vitro*, the equation (1) cannot be used anymore and other method to analyse the growth, such as fractal scaling technique, has to be used [29],[30].

Fulfilling the above conditions, the DP time of treated cells is 59 h respect to 44 h of the control. From these results we demonstrate that the synergic action of potassium bicarbonate and D-ribose has effect on Hs 578T cancer cell line proliferation reducing the cell cycle time of 5 mM K:D-rib treated cells by 30% respect to the control.

### Chemotactic invasion assay

In order to demonstrate the effects of K:D-rib water solution at the concentration of 5 mM and how it can modify the tumorigenic potential of human breast cancer cell line Hs 578T, we performed the chemotactic invasion assay. This assay gives the information about the invasive potential of cancer cells under chemotactic stimuli provide by FBS (fetal bovine serum). Because Hs 578T cells interact with the AGAR – FBS spot and invade it, this assay fits together migration and invasion, two important hallmarks of the cancer cells.

Hs 578T cell line is able to migrate across the coverslip toward the FBS – agar spot and to invade it within 48 hours (Figure 3 - A) in any directions not privileging anyone respect to other. We can assume that the spot gets an equal physical resistance to cell invasion. On the contrary the AGAR spot without FBS does not exert the same stimuli to Hs 578 T cell line, which accumulate on the spot edge and do not get into the spot (Figure 3 - C).

We have investigated how K:D-rib solution, at the concentration of 5 mM modifies the cancer features mentioned above. In the Figure 4 the relative cell number inside the AGAR-FBS drops down already after five days of treatment. After nine days of treatment the cells are almost not able to invade the AGAR – FBS spot, demonstrating that tumorigenic potential highly decreases at the ninth day of the treatment. The difference of relative cell number between the seven days of the treatment and the nine days one is the highest, around 30%. These results show that 5 mM K:D-rib interferes *in vitro* with the capability of Hs 578T cell line to migrate under chemotactic stimuli, to reach the stimuli source and to invade a different texture structure.

After 7 days of treatment, the chemotactic invasion assay was made both prolonging and suspending the treatment during the assay. Hs 578T cell line pre-treated for seven days and assayed for 48 h without treatment shows comparable values with those obtained from the fifth day of the pre-treatment and assayed for 48 h with treatment.

These results drive us to believe the K:D-rib solution as modifier of tumorigenic marks even if it is employed during the assay.

Another aspect that it should be considered is the tracks inside AGAR-FBS spot. The cancer cells after 9 days of treatment show a shorter path, that demonstrates difficulties for migration and invasion, two important cancer cell hallmarks changed by K:D-rib treatment.

### Hs 578Bst cell line

The effects of K:D-rib solution on cell growth rate are detectable counting the splitting number during the experimental extent. This type proliferation assessment has been used, because of the unreliability of the broadly used metabolic assays as MTT assay. These results recently published, prove the interaction of MTT salt (tetrazolium bromide) with the K:D-rib solution and the solution reduces MTT salt to formazan as the ascorbic acid does [12],[27]. The splitting number is a useful cell culture laboratory practice, which gives information about cells trend growth. We have used this tool to assess the K:D-rib effects on proliferation of non-tumor Hs 578Bst cell line. Hs 578Bst cells treated for 19 days, with K:D-rib solution at the concentration of 5 mM, do not show toxicity and or noteworthy cell proliferation slowing rate. The splitting number of the treated and untreated cells is the same. Comparing the effects of K:D-rib solution on proliferation of Hs 578T cell line (human breast cancer) and Hs 578Bst cell line (human breast epithelial) is evident that the magnitude effect is cell type specific. K:D-rib dampens the Hs 578T cell line proliferation rate already within the first treatment week. Treated Hs 578Bst cells do not show any duplication cell rate slowing during the treatment period.

The data of the chemoinvasion assay and the results from the cancer and not cancer cell proliferation demonstrate that the 5 mM K:D-rib water solution is a powerful inhibitor of cancer potential and a strong antiproliferative agent. Despite this, K:D-rib solution, does not exhibit any appreciable toxicity as confirmed by the proliferation assay accomplished on Hs 578Bst cell line.

## Competing interest

LB had a INBB grant supported by Valsè Pantellini Foundation that has specific interest in the study of Potassium Bicarbonate and D-Ribose compounds.

AAB, IO; SC declare that they have no competing interests.

## Authors’ contributions

LB contributed in the experimental design, carried out the experiments with particular reference of Chemotactic invasion assay. AAB was involved in the cell counting using the ImageJ program and results discussion. IO contributed to results discussion and manuscript revision. The SC analyzed and interpreted the data, and contributed in drafting and manuscript revision. All of the authors read the manuscript, contributed in correcting it and approving its final version.
